# Prevalence of *Enterocytozoon bieneusi* and *Blastocystis* sp. infection in foxes (*Alopex lagopus*) in northern China

**DOI:** 10.1051/parasite/2025010

**Published:** 2025-02-24

**Authors:** Yan Tang, Hai-Tao Wang, Xue-Min Li, Zhong-Yuan Li, Qing-Yu Hou, Jing Jiang, Li-Hua Yang, Ya Qin

**Affiliations:** 1 College of Pharmacy, Guizhou University of Traditional Chinese Medicine Guiyang 550025 Guizhou Province PR China; 2 College of Life Sciences, Changchun Sci-Tech University Shuangyang 130600 Jilin Province PR China; 3 College of Veterinary Medicine, Qingdao Agricultural University Qingdao 266109 Shandong Province PR China; 4 Guangxi Key Laboratory of Brain and Cognitive Neuroscience, College of Basic Medicine, Guilin Medical University Guilin 541004, Guangxi Zhuang Autonomous Region PR China; 5 College of Animal Science and Technology, Jilin Agricultural University Changchun 130118 Jilin Province PR China

**Keywords:** Microeukaryote, Genotypes, Subtypes, Zoonosis

## Abstract

To examine the prevalence of *E. bieneusi* and *Blastocystis* sp. in foxes in China, this study analyzed the prevalence and distribution of genotypes or subtypes. A total of 352 fresh fecal samples were collected from foxes across five provinces in northern China and analyzed using PCR. The overall prevalences of *E. bieneusi and Blastocystis* sp. were 48.3% and 2.0%, respectively; the highest prevalences were found in Shandong Province, with 87.1% and 5.4%, respectively. The prevalence rates were influenced by several factors; a breeding scale value <1,500 was related to higher prevalences. Multivariate analysis showed that the region and breeding scale were the main risk factors for *E. bieneusi*. Eleven genotypes of *E. bieneusi* were identified, all of which are classified within Group 1. This includes five previously characterized genotypes and six novel genotypes. Among these, CHN-F1 was the predominant genotype, accounting for 67.7% of cases. *Blastocystis* sp. was detected with only one subtype (ST3), which represents the first report of this genotype in foxes. The identification of *E. bieneusi* in foxes and the first detection of the ST3 subtypes of *Blastocystis* sp. contribute to a more comprehensive understanding of the epidemiology of these microeukaryotes. These findings suggest a potential pathway for the transmission of microeukaryotes from fox farms to human populations, underscoring the importance of monitoring the public health risks.

## Abstract (fr)

Pour examiner la prévalence d’*E. bieneusi* et de *Blastocystis* sp. chez les renards en Chine, cette étude a analysé la prévalence et la distribution des génotypes ou sous-types. Au total, 352 échantillons de matières fécales fraîches ont été collectés auprès de renards dans cinq provinces du nord de la Chine et analysés par PCR. Les prévalences globales d’*E. bieneusi* et de *Blastocystis* sp. étaient respectivement de 48,3 % et de 2,0 %; les prévalences les plus élevées ont été trouvées dans la province du Shandong, avec respectivement 87,1 % et 5,4 %. Les taux de prévalence étaient influencés par plusieurs facteurs; une valeur d’échelle de reproduction < 1500 était liée à des prévalences plus élevées. Une analyse multivariée a montré que la région et l’échelle de reproduction étaient les principaux facteurs de risque pour *E. bieneusi*. Onze génotypes d’*E. bieneusi* ont été identifiés, tous classés dans le groupe 1. Ils incluent cinq génotypes précédemment caractérisés et six nouveaux génotypes. Parmi ceux-ci, le génotype CHN-F1 était prédominant, représentant 67,7 % des cas. *Blastocystis* sp. a été détecté avec un seul sous-type (ST3), ce qui représente le premier signalement de ce génotype chez les renards. L’identification d’E*. bieneusi* chez les renards et la première détection des sous-types ST3 de *Blastocystis* sp. contribuent à une compréhension plus complète de l’épidémiologie de ces microeucaryotes. Ces résultats suggèrent une voie potentielle de transmission des microeucaryotes des élevages de renards aux populations humaines, soulignant l’importance de surveiller les risques pour la santé publique.

## Introduction

*Enterocytozoon bieneusi* and *Blastocystis* sp. are prevalent microeukaryotes [18, 33, 40, 67]. They can infect various hosts, including humans and foxes. Humans and animals become infected with *E. bieneusi* and *Blastocystis* sp. mainly through the fecal–oral ingestion of contaminated water or food [57]. Infections with two microeukaryotes are usually asymptomatic, but they can sometimes cause diarrhea [32, 67].

To date, at least 820 genotypes of *E. bieneusi* and 48 subtypes (STs) of *Blastocystis* sp. have been identified [32, 36, 39, 40, 54]. The *E. bieneusi* genotypes have been divided into 15 phylogenetic groups with different host specificity and zoonotic potential [32]. Group 1 is the largest, containing numerous zoonotic genotypes like D, Ebpc, and Peru 8, which are frequently detected in humans [24]. Even though certain genotypes like CHN-F1, NCF2, and NCF3 within Group 1 have not been detected in human patients thus far, they may still pose a zoonotic transmission risk [8, 32, 44]. The genotypes in Group 2 primarily infect ruminants, but also include the BEB4, BEB6, I, and J genotypes capable of infecting humans [36]. The remaining Groups 3–15 may exhibit host specificity, with some genotypes reported to have the ability to infect humans, such as Group 5 (KIN-3) and Group 6 (Nig 3 and May1) [15, 23]. More than one billion individuals worldwide are carriers of *Blastocystis* sp., with human infections involving ST1-10, ST12, ST14, ST16, ST23, ST35, and ST41 among the 48 identified subtypes. The prevalence of *Blastocystis* sp. infection in humans is predominantly attributed to ST1–4 which accounts for approximately 90.0% of cases [4, 21, 25, 29, 42, 65]. Although there are already reports on the prevalence of the *E. bieneusi* genotypes and the *Blastocystis* sp. subtypes, additional research is necessary to comprehensively understand their implications for public health [42].

A total of 361 *E. bieneusi* genotypes have been identified in China, including a variety of hosts, such as humans (5.7%, 188/3271, predominantly genotypes D and Ebpc), non-human primates (17.8%, 948/5318, predominantly genotype D), cattle (14.0%, 783/5594, predominantly genotypes J and I), sheep and goats (31.9%, 978/3068, predominantly genotype BEB6), pigs (55.9%, 1101/1969, predominantly genotypes Ebpc and EbpA), and blue foxes (17.9%, 108/603, predominantly genotype D) [53]. *Blastocystis* sp. is also widely distributed in China, the average infection rate among humans is 3.4% (3625/107,695), with ST3 being the predominant genotype [10]. Among other animals, the overall infection rate in Artiodactyla (including pigs, cattle, sheep, goats, reindeer, and sika and barking deer) is 27.4% (1050/3828), while in Carnivora (including raccoon domestic dogs and Arctic foxes), the infection rate is 2.8% (11/389) [10]. Another study involved a meta-analysis, showing that the global prevalence of *Blastocystis* sp*.* was 2.1% (29/1377) in free-living carnivores, compared to a significantly higher prevalence of 8.5% (100/1175) in captive individuals [5].

In China, the fox is an economically important animal, with approximately 3.44 million breeding foxes [66]. However, there is limited research on the prevalence of *E. bieneusi* and *Blastocystis* sp. in foxes, as well as their genetic characteristics. This study aimed to investigate the infection and distribution of the *E. bieneusi* genotypes and the *Blastocystis* sp. subtypes in foxes in China. Understanding the genetic evolutionary relationship between *E. bieneusi* and *Blastocystis* sp. in foxes can help us to effectively prevent and control diseases among fox populations as well as safeguard public health in China.

## Materials and methods

### Ethics statement

All the procedures used in this study were approved by the Research Ethics Committee for the Care and Use of Laboratory Animals in Qingdao Agricultural University, China. Written informed consent was obtained from the owners of the animals involved in this study.

### Collection of samples

From October 2023 to June 2024, a total of 352 feces samples from foxes (*Alopex lagopus*) were collected across five provinces in northern China. Specifically, the samples were collected from three farms in Heilongjiang (sample number = 47, taken in 2024), two farms in Jilin (sample number = 40, taken in 2024), six farms in Liaoning (sample number = 10, taken in 2023; sample number = 69, taken in 2024), six farms in Hebei (sample number = 93, taken in 2024), and five farms in Shandong (sample number = 93, taken in 2023). Fresh fecal samples, representing approximately 1.0 to 1.5% of the total farmed population, were randomly collected from each farm. All the farms underwent biannual deworming treatments using either ivermectin or albendazole. Diarrhea is defined as the presence of green, watery, or bloody stools. The samples were collected from multiple locations within the farms to avoid being limited to a single area or cage. During the collection process, samples that met the defined criteria for diarrhea were recorded as diarrheal samples. The sampling process involved recording essential information, including the region, the breeding scale, the age of the fox, and the presence of diarrhea, which were documented during the data collection process. The collected samples were transported to a laboratory on ice within 24–48 h and stored at −80 °C until DNA extraction. The storage duration ranged from 1 week to 10 months.

### DNA extraction and PCR amplification

DNA extraction from all the samples was conducted utilizing an EZNAR Stool DNA Kit (OMEGA Biotek Inc., Norcross, GA, USA), with the material subsequently stored at −20 °C. The DNA of all the samples was amplified using PCR to determine the presence of *E. bieneusi*, or *Blastocystis* sp., based on the ITS region and the small subunit (*SSU*) rRNA gene [57, 59], respectively. Novoprotein 2×Taq Master Mix (Novoprotein Ltd., E005-01, Suzhou, China) was used for all the PCR amplifications. The reaction conditions and sizes of the product fragments are presented in Table 1. *Enterocytozoon bieneusi* (genotype D) and *Blastocystis* sp*.* (subtype 3)-positive DNA previously detected in a laboratory from wild rodents was used as the positive control, while dd H_2_O was employed as the negative control [15, 16]. The PCR products were visualized using the QuickGel 6200 gel imaging System (Mona Biotechnology Co., Ltd., GD50102, Suzhou, China) after electrophoresis on 1% agarose gels.

**Table 1:** Primers used in the characterization of *E. bieneusi* and *Blastocystis* sp.

Gene	Primer (5′–3′)	Product size (bp)	Annealing temperature (°C)
SSU rRNA of Blastocystis sp.	SSU rRNA-F: ATCTGGTTGATCCTGCCAGT	600	55
	SSU rRNA-R: GAGCTTTTTAACTGCAACAACG		
ITS gene of E. bieneusi	ITS-F1: GGTCATAGGGATGAAGAG	392	57
	ITS-R1: TTCGAGTTCTTTCGCGCTC		
	ITS-F2: GCTCTGAATATCTATGGCT		55
	ITS-R2: ATCGCCGACGGATCCAAGTG		

### Sequencing and phylogenetic analyses

All amplicons with the expected size were sequenced bidirectionally at the General Biol. Company in Anhui, China. Then, the obtained sequences were blasted in GenBank to finally confirm the positive samples (http://www.ncbi.nlm.nih.gov/BLAST/). The genotypes of *E. bieneusi* were determined and named based on ~243 bp of the ITS region, according to the established nomenclature system [38]. Phylogenetic trees were constructed using the neighbor-joining (NJ) method in MEGA 11 (v. 11.0.13), with bootstrapping performed using 1,000 replicates to assess the genetic relationships among the *E. bieneusi* genotypes. CD-HIT (Version 4.8.1) was employed to remove the redundant sequences, solely preserving the unique sequences at a 100% identity threshold. The unique sequences, which serve as representatives of their respective clusters, were subsequently submitted to GenBank (accession Nos. PQ249072–PQ249088 and PQ223428–PQ223432).

### Statistical analysis

The chi-square test of SAS (SAS Institute Inc., Cary, NC, USA, v.9.4) was utilized to conduct statistical analysis of the region, the breeding scale, the age of the foxes, and the presence of diarrhea. Forward stepwise logistic regression, using the Fisher scoring technique, was applied to a binary Logit model to assess the impact of multivariate regression analysis on *E. bieneusi* and *Blastocystis* sp. infection. All the tests were two-sided, and the results were considered statistically significant if *p* < 0.05, with corresponding odds ratios (ORs) and their 95% confidence intervals (95% CIs) provided [63].

## Results

### Prevalence of *Enterocytozoon bieneusi* and *Blastocystis* sp.

This study identified a prevalence of 48.3% (170/352, 95% CI [42.97–53.65]) for *Enterocytozoon bieneusi* and 2.0% (7/352, 95% CI [0.80–4.05]) for *Blastocystis* sp. Among the 352 foxes examined (Tables 2 and 3). *Enterocytozoon bieneusi* was detected across all five provinces, with the highest prevalence observed in Shandong (87.1%, 81/93, 95% CI [79.43–93.24]), followed by Liaoning (65.8%, 52/79, 95% CI [54.96–75.93]), Jilin (27.5%, 11/40, 95% CI [14.61–42.51]), Hebei (20.4%, 19/93, 95% CI [12.79–29.28]), and Heilongjiang Province (14.9%, 7/47, 95% CI [5.93–26.70]) (Table 2). The occurrence of *Blastocystis* sp. was relatively low, being detected solely in Shandong and Hebei Provinces, with infection rates of 5.4% (5/93, 95% CI [1.54–11.06]) and 2.2% (2/93, 95% CI [0.03–6.37]), respectively (Table 3).

**Table 2:** Factors associated with prevalence of *E. bieneusi* in foxes.

Factor	Category	No. positive/tested	% (95% CI*)	Univariate meta-regression		Multivariate analysis^c^	
				OR (95% CI)	*p*-Value	OR (95% CI)	*p*-Value
Region	Heilongjiang Province	7/47	14.9 (5.93–26.70)	Reference^d^	<0.0001	0.408 (0.339–0.491)	<0.0001
	Jilin Province	11/40	27.5 (14.61–42.51)	2.17 (0.75–6.27)			
	Liaoning Province	52/79	65.8 (54.96–75.93)	11.05 (4.35–27.83)			
	Hebei Province	19/93	20.4 (12.79–29.28)	1.47 (0.57–3.79)			
	Shandong Province	81/93	87.1 (79.43–93.24)	39.52 (2.95–11.67)			
Breeding scale	<1500	83/153	54.3 (46.30–62.10)	1.53 (1.00–2.33)	0.0506	0.468 (0.275–0.796)	<0.0001
	≥1500	87/199	43.7 (36.88–50.68)	Reference			
Diarrhea	Yes	45/74	60.8 (49.39–71.68)	1.90 (1.13–3.21)	0.0156	–	–
	No	125/278	45.0 (39.15–50.85)	Reference			
Age of Fox	Adult^a^	53/182	29.1 (22.73–35.95)	Reference	<0.0001	–	–
	Juvenile^b^	117/170	68.8 (61.64–75.59)	12.38 (7.15–21.45)			
Total		170/352	48.3 (42.97–53.65)				

CI*: confidence interval; a: ≥1 year old; b: <1 year old; c: Only effect factors are listed; d: control group for risk analysis.

**Table 3:** Factors associated with prevalence and subtypes of *Blastocystis* sp. in foxes.

Factor	Category	No. positive/tested	% (95% CI*)	Univariate meta-regression	
				*p*-Value	OR (95% CI)
Region	Heilongjiang Province	0/47	0 (0.00–3.63)	0.0927	–
	Jilin Province	0/40	0 (0.00–4.25)		–
	Liaoning Province	0/79	0 (0.00–2.16)		–
	Hebei Province	2/93	2.2 (0.03–6.37)		Reference^c^
	Shandong Province	5/93	5.4 (1.54–11.06)		2.59 (0.49–13.68)
Breeding scale	<1500	5/153	3.3 (0.93–6.79)	0.1439	3.33 (0.64–17.40)
	≥1500	2/199	1.0 (0.02–3.01)		Reference
Diarrhea	Yes	3/74	4.1 (0.51–10.02)	0.1673	Reference
	No	4/278	1.4 (0.31–3.25)		2.89 (0.63–13.23)
Age of Fox	Adult^a^	2/182	1.1 (0.02–3.28)	0.2323	Reference
	Juvenile^b^	5/170	2.9 (0.83–6.12)		2.73 (0.52–14.25)
Total		7/352	2.0 (0.80–4.05)		

CI*: confidence interval; a: ≥1 year old; b: <1 year old; c: control group for risk analysis.

Statistical analysis revealed that the infection rate of *E. bieneusi* was significantly higher in foxes with a breeding scale value of less than 1500 (54.3%, 83/153, 95% CI [43.60–62.10]) compared to those with a breeding scale value greater than 1500 (43.7%, 87/199, 95% CI 36.88–50.68) (*p* < 0.0001). Notably, the prevalence of *E. bieneusi* was 60.8% in diarrheal foxes, which is significantly higher than that in non-diarrheal foxes (45.0%, 125/278, 95% CI [39.15–50.85]) (*p* = 0.0156). Additionally, the prevalence in juvenile foxes (68.8%, 117/170, 95% CI [61.64–75.59]) was significantly higher than that in adult foxes (29.1%, 53/182, 95% CI [22.73–35.95]) (*p* < 0.001, Table 2).

Similarly, the infection rate of *Blastocystis* sp. was higher in foxes with a breeding scale value of less than 1500 (3.3%, 5/153, 95% CI [0.93–6.79]) compared to those with a breeding scale value greater than 1500 (1.0%, 2/199, 95% CI [0.02–3.01]) (*p* = 0.0927). The prevalence of *Blastocystis* sp. was 4.1% (3/74, 95% CI [0.51–10.02]) in diarrheal foxes, which is higher than in non-diarrheal foxes (1.4%, 4/278, 95% CI [0.31–3.25]) (*p* = 0.1673). Furthermore, the prevalence in juvenile foxes (2.9%, 5/170, 95% CI [0.83–6.12]) was significantly higher than that in adult foxes (1.1%, 2/182, 95% CI [0.02–3.28]) (*p* = 0.2323, Table 3).

### Risk factors of *E. bieneusi* and *Blastocystis* sp.

The region and breeding scale were significant in the final model, indicating its strong influence on *E. bieneusi*. The region and breeding scale of foxes had a strong effect on *E. bieneusi* infection in this study. The foxes in Shandong Province (OR = 0.408, 95% CI [0.339–0.491]) appear to exhibit a higher susceptibility to *E. bieneusi* compared to those in Liaoning, Heilongjiang, Jilin, and Hebei Provinces. A breeding scale value < 1500 (OR = 0.468, 95% CI [0.275–0.796]) indicates greater susceptibility to *E. bieneusi* than a value ≥ 1500. No significant influencing factors were identified in the presence of *Blastocystis* sp. in the foxes.

### Distribution of *E. bieneusi* genotypes and *Blastocystis* sp. subtypes

**Figure 1:**
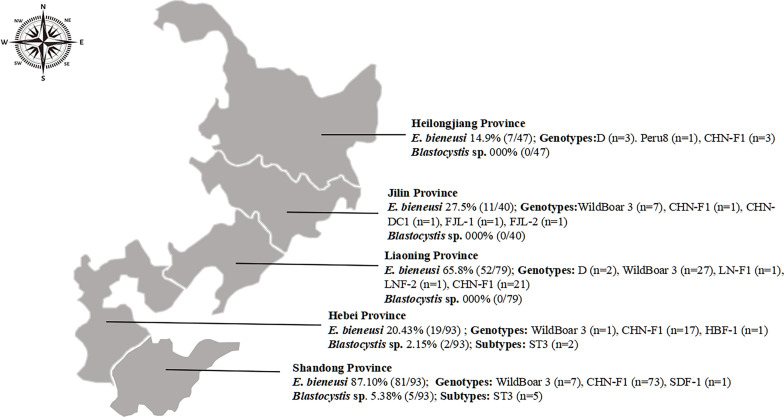
Distribution of *E. bieneusi* genotypes and *Blastocystis* sp*.* subtypes.

This study identified eleven genotypes of *E. bieneusi* and one subtype of *Blastocystis* sp. among the 352 fox samples. The genotypes of *E. bieneusi* comprise five known (D, Peru 8, WildBoar 3, CHN-F1, and CHN-DC1) and six newly identified genotypes (LNF-1, LNF-2, HBF-1, SDF-1, FJL-1, and FJL-2). These new genotypes contain a total of 11 polymorphic sites (Table 4). In this study, the recently discovered genotype CHN-F1 exhibited the highest prevalence rate, followed by NCF3, NCF2, and D. Type D was detected in Heilongjiang and Liaoning Provinces. Notably, CHN-F1 was detected in all the five provinces, whereas the remaining new genotypes LNF-1 and LNF-2 were found only in Liaoning Province, SDF-1 was detected in Shandong Province, and HBF-1 was found in Hebei Province. The ST3 subtype of *Blastocystis* sp. was exclusively detected in this study, limited to Hebei and Shandong Provinces (Fig. 1).

**Table 4:** Variations in the ~243 ITS nucleotide sequences among genotypes of the *E. bieneusi* in foxes.

Genotypes (No.)	Nucleotide at position											GenBank accession No.
	17	44	48	65	77	93	117	131	191	209	212	
D (Reference)	G	T	T	G	G	C	T	G	G	T	G	AF023245 (Intergenic spacer)
D (5)												PQ249073
CHN-DC1 (1)				A		T	G	A				PQ249076
FJL-1 (1)	A	C		A		T	G	A				PQ249075
CHN-F1 (115)	A			A		T	G	A				PQ249072
HBF-1 (1)	A			A		T	G	A			A	PQ249087
LN-F1 (1)					A							PQ249080
LNF-2 (1)									A	C		PQ249082
FJL-2 (1)			C			T	G	A				PQ249077
WildBoar 3 (42)						T	G	A				PQ249079
Peru8 (1)							G					PQ249074
SDF-1 (1)	A						G	A				PQ249083

### Phylogenetic analysis

**Figure 2:**
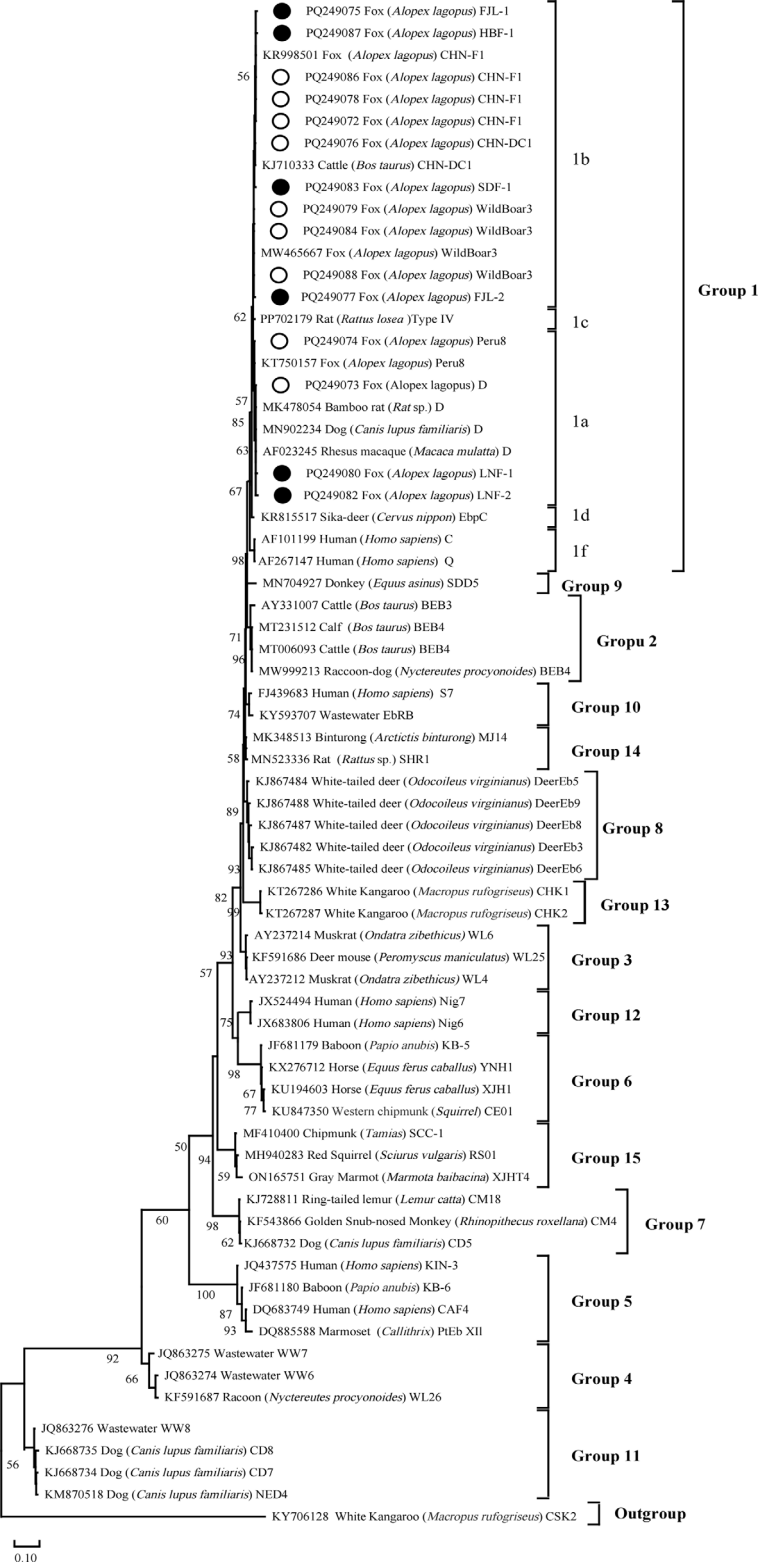
Neighbor-joining tree of *E. bieneusi* ITS genotypes. Phylogenetic relationships of ITS nucleotide sequences of the *E. bieneusi* genotypes identified in this study and other reported genotypes. The phylogeny was inferred by a neighbor-joining analysis. Bootstrap values were obtained using 1000 replicates; those with values >50% are shown on the nodes. The genotypes in this study are marked by empty circles, and the novel genotypes are marked by filled circles.

Phylogenetic trees were constructed to represent the eleven *E. bieneusi* genotypes (Fig. 2) and the one *Blastocystis* sp. subtype identified in this study. It was observed that all the eleven *E. bieneusi* genotypes were grouped in Group 1, with D, Peru8, and the novel genotypes LNF-1 and LNF-2 classified within Group 1a. The remaining detected genotypes CHN-F1, CHN-DC1, and WildBoar 3, as well as the newly discovered genotypes FJL-1, SDF-1, FJL-2, and HBF-1, were classified into Group 1b.

## Discussion

*Enterocytozoon bieneusi* and *Blastocystis* sp. are significant zoonotic intestinal protozoa that have been understudied in foxes [1]. This study employed molecular epidemiologic methods to investigate the prevalence of these protozoa among foxes in northeastern China. Additionally, this study characterized the predominant *E. bieneusi* genotypes and *Blastocystis* sp. subtypes in foxes and evaluated the potential zoonotic risk.

The prevalence of *E. bieneusi* was 48.3% (170/352), significantly higher than that reported in previous studies in foxes in China, such as 27.7% (53/191) [55], 16.4% (18/110) [66], and 12.3% (37/302) [62]. These studies employed the same PCR method as in the current study. In this study, the prevalence rate of *Blastocystis* sp. was 2.0%. Other studies have reported that the prevalence of *Blastocystis* sp. in foxes is 1.9% (4/213) in China [46] and 2.2% (4/213) in Spain [6]. These findings are consistent with the prevalence observed in our study. Strict carnivores tend to exhibit a lower prevalence of *Blastocystis* sp. than omnivorous or herbivorous species, and *Blastocystis* sp. in foxes may be caused by the contamination of food or water sources [5, 45].

Regional subgroup analysis revealed significant variation in *E. bieneusi* prevalence across the five provinces (*p* < 0.05). The prevalence in this study is much higher than that in previous studies, such as in Jilin (7.7%) [62], Heilongjiang (7.1–27.7%) [55, 62], Hebei (17.7%) [62], and Xinjiang (2.9%) [64]. The previous studies reported 1.9% *Blastocystis* sp. prevalence in Jilin and Liaoning Provinces [46]. In comparison, the prevalence in Shandong and Hebei Provinces was higher than that given in previous reports. This study presents the first epidemiologic survey on *Blastocystis* sp. in foxes conducted in Hebei Province. Although it exhibited a low positive rate, this should still raise concerns among individuals. In provinces with a high prevalence of both *E. bieneusi* and *Blastocystis* sp., it is important to monitor these microeukaryotes, although their veterinary significance and impact on foxes remain unclear. This includes ensuring strict adherence to environmental hygiene practices on farms and regular disinfection protocols, as well as routine testing. Furthermore, this study revealed a correlation between the breeding scale and the prevalence of these two protists, with farms having a breeding scale value <1500 exhibiting higher prevalence than those with a breeding scale value ≥1500. This could be attributed to the fact that large-scale farms possess greater expertise in feeding management and disease prevention and control [52].

In this study, the infection rate of *E. bieneusi* was higher in the diarrheal foxes than it was in the non-diarrheal samples (*p* < 0.05); a similar observation has been made in humans and cattle [36, 61]. Among patients exhibiting various degrees of diarrhea, the prevalence of *E. bieneusi* is notably higher compared to that among individuals without gastrointestinal (GI) disorders [3, 10, 60]. This may be because *E. bieneusi* can indeed cause diarrhea in the host, particularly under conditions of self-limiting infection or impaired host immunity [32]. Although the infection rate of *Blastocystis* sp. was higher in the diarrheal samples than it was in the non-diarrheal samples, the difference was not statistically significant (*p* > 0.05).

Both *E. bieneusi* and *Blastocystis* sp. exhibited higher infection rates in juvenile foxes compared to those of the adult foxes. The findings of this study are consistent with prior research, indicating that juvenile foxes are more vulnerable to infection [8, 44]. Further epidemiological data and laboratory studies on their pathogenic mechanisms may be necessary to provide a scientific explanation for the differing infection rates of these two protozoa species in juvenile and adult foxes.

Eleven genotypes of *E. bieneusi* belonging to Group 1 were identified in the foxes in this study, including five known (D, Peru8, WildBoar 3, CHN-F1, and CHN-DC1) and six newly discovered genotypes (LNF-1, LNF-2, HBF-1, SDF-1, FJL-1, and FJL-2). Genotype D exhibits a strong capacity for cross-species transmission [14], and has been reported in humans in China, highlighting the need for vigilance regarding the potential transmission risk from foxes to humans [27, 35, 49, 50]. Additionally, the genotype Peru 8 has been reported to infect humans in other studies [2, 7]. Although only one Peru8-positive sample was found in this study, this genotype has been consistently detected in foxes, suggesting that they are susceptible hosts for genotype Peru 8 [62]. Notably, genotype CHN-F1 was predominant, found in all the subgroups, with a prevalence of 67.7% (115/170). First identified in dairy cattle in 2015, CHN-F1 has also been detected in raccoon dogs and has exhibited high prevalence rates in foxes in Henan Province (52.1%, 25/48) [44, 55, 66]. Aside from China, only two reports from Europe indicate that CHN-F1 can infect pigeons and chinchillas, suggesting its potential for global spread [22, 68]. The prevalence rates of WildBoar 3 and CHN-DC1 among the positive samples were 24.7% (42/170) and 0.6% (1/170), respectively. WildBoar 3 exhibits a broad spectrum of host adaptability and has been documented in wild boars from Poland, Czechia [30], and Spain [9], as well as in red foxes and stone martens from Portugal [13], and in goats from China [11]. Other reports confirm their presence in foxes, indicating their common occurrence [8, 62]. Phylogenetic analysis identified six new genotypes of *E. bieneusi*. Group 1 is recognized for its zoonotic potential, indicating that these six novel genotypes may have the capacity for cross-host transmission [20]. Therefore, it is crucial to maintain ongoing attention to these genotypes in future investigations. Consequently, individuals such as fox breeders and those with weakened immunity – such as patients with HIV, children, and the elderly – should be especially vigilant regarding the risk of *E. bieneusi* transmission from foxes.

Only the ST3 subtype was detected in *Blastocystis* sp. in this study. This study was the first to identify the ST3 subtype in foxes. Previous studies have reported the presence of the ST1, ST4, and ST7 subtypes in foxes from northeast China [46], while foxes in Spain were found to harbor the ST7 and ST14 subtypes [6]. ST3 is the most common genotype in humans and the predominant subtype of *Blastocystis* sp. infections in China, with prevalence rates ranging from 43.8% to 62.0% [10, 31, 34, 37]. Another study has shown that the sequences of ST3 subtypes in dairy cows are identical to those found in exposed humans [17], suggesting a potential risk of ST3 cross-species transmission between humans and animals. It should be noted that there is 99.7% homology between the ST3 subtype (PQ223430) studied here and that in humans (MK782518). This indicates potential for the transmission of *Blastocystis* sp. between foxes and humans. However, additional follow-up studies are required to confirm this hypothesis. Other researchers should concurrently collect fecal samples from both feeders and foxes during the sampling process to determine whether such transmission is possible. ST3 is also widely prevalent among various animal species in China, such as rodents [48], wild rhesus macaques [56], Pallas’s squirrels (*Callosciurus erythraeus*) [28], pet dogs [26], rabbits [58], pigs [19, 51], pigeons [43], raccoon dogs [46], and beef [47] and dairy cows [12, 41]. Given that ST3 is widely distributed in both humans and a variety of animals in China, it is important to continue investigating the ecological dynamics and transmission routes of *Blastocystis* sp.

This study investigated the prevalence of *E. bieneusi* genotypes and *Blastocystis* sp. subtypes in Chinese foxes, providing compelling evidence for the occurrence of infection in this particular host species. However, it is important to acknowledge certain limitations associated with this research. The sampling scope was confined to northeast China, potentially limiting the generalizability of the prevalence rates of *E. bieneusi* and *Blastocystis* sp. in foxes across China. Additionally, this study did not account for variations among fox breeds, which could influence the infection status and genetic distribution of these protists. Therefore, future research should broaden the sampling area and increase the sample size, while also meticulously documenting relevant sample information.

## Conclusion

There was a significant prevalence of *E. bieneusi* among foxes, with genotype CHN-F1 being widely distributed. Additionally, foxes carried genotypes D and Peru 8, which infect humans. Furthermore, new genotypes of *E. bieneusi* (LNF-1, LNF-2, SDF-1, HBF-1, FJL-1, and FJL-2) were identified for the first time, all belonging to Group 1 with a potential zoonotic risk. ST3 was found to be the predominant subtype responsible for human *Blastocystis* sp. infection in China. Moreover, this study provides the first evidence that foxes serve as carriers of ST3. Consequently, foxes represent a plausible source of transmission for both *E. bieneusi* and *Blastocystis* sp. infection. This investigation contributes to our understanding of the prevalence and genetic diversity of *E. bieneusi* and *Blastocystis* sp. among the fox populations of China, while highlighting its public health significance in preventing potential zoonotic transmission mediated by foxes.

## Data Availability

The gene sequences obtained in this study have been submitted to GenBank (accession Nos. PQ249072–PQ249088, and PQ223428–PQ223432).
